# Palliativversorgung am Lebensende in Deutschland: Inanspruchnahme und regionale Verteilung

**DOI:** 10.1007/s00103-020-03240-6

**Published:** 2020-11-13

**Authors:** Bianka Ditscheid, Markus Krause, Thomas Lehmann, Kathleen Stichling, Maximiliane Jansky, Friedemann Nauck, Ulrich Wedding, Werner Schneider, Ursula Marschall, Winfried Meißner, Antje Freytag, die SAVOIR-Studiengruppe

**Affiliations:** 1 Institut für Allgemeinmedizin, Universitätsklinikum Jena, Bachstr. 18, 07743 Jena, Deutschland; 2 Zentrum für Klinische Studien, Universitätsklinikum Jena, Jena, Deutschland; 3 Klinik für Palliativmedizin, Universitätsmedizin Göttingen, Göttingen, Deutschland; 4 Abteilung Palliativmedizin der Klinik für Innere Medizin II, Universitätsklinikum Jena, Jena, Deutschland; 5 Zentrum für Interdisziplinäre Gesundheitsforschung, Universität Augsburg, Augsburg, Deutschland; 6 BARMER, Wuppertal, Deutschland

**Keywords:** Ambulante Palliativversorgung, AAPV, SAPV, GKV-Routinedaten, Regionale Unterschiede, Palliative homecare, PPC, SPHC, Claims data, Regional comparison

## Abstract

**Hintergrund:**

Das Angebot an Palliativversorgung hat in Deutschland stark zugenommen. Weitgehend unbekannt ist, wie viele Menschen am Lebensende welche palliativen Versorgungsformen in Anspruch nehmen und welche regionalen Unterschiede bestehen.

**Methode:**

Retrospektive Kohortenstudie mit GKV-Routinedaten (BARMER) über Versicherte mit Sterbedatum im Jahr 2016: Anhand einer mindestens einmalig abgerechneten Leistung in den letzten 6 Lebensmonaten wurde die Inanspruchnahme allgemeiner ambulanter Palliativversorgung (AAPV), spezialisierter ambulanter Palliativversorgung (SAPV) sowie stationärer Palliativ- und Hospizversorgung ermittelt. Erstmals wurden auch Abrechnungsziffern kassenärztlicher Vereinigungen und selektivvertragliche Sonderziffern für palliativmedizinische Leistungen sowie SAPV-Leistungsabrechnungen herangezogen.

**Ergebnisse:**

Von den 95.962 Verstorbenen der Studienpopulation wurden bundesdurchschnittlich 32,7 % palliativ versorgt, mit Schwankungen zwischen 26,4 % in Bremen und 40,8 % in Bayern. AAPV-Leistungen wurden bei 24,4 % abgerechnet (16,9 % in Brandenburg bis 34,1 % in Bayern). SAPV-Leistungen erhielten 13,1 % (6,3 % in Rheinland-Pfalz bis 18,9 % in Brandenburg bzw. 22,9 % in Westfalen-Lippe mit abweichender SAPV-Praxis). Stationär palliativmedizinisch versorgt wurden 8,1 % (6,7 % in Schleswig-Holstein/Hessen bis 13,0 % in Thüringen), Hospizleistungen wurden für 3,3 % abgerechnet (1,6 % in Bremen bis 5,6 % in Berlin).

**Diskussion:**

SAPV wird häufiger in Anspruch genommen als bisher berichtet, AAPV ist rückläufig. Die jeweilige Inanspruchnahme scheint weniger durch objektiven Bedarf als durch regionalspezifische Rahmenbedingungen begründet. Die Weiterentwicklung der Palliativversorgung sollte zukünftig neben Bedarfskriterien mehr an Outcomes sowie dafür relevanten Rahmenbedingungen orientiert werden.

**Zusatzmaterial online:**

Zusätzliche Informationen sind in der Online-Version dieses Artikels (10.1007/s00103-020-03240-6) enthalten.

## Abstract (en)

**Background:**

Palliative care supply increased in Germany in recent years. But how many people use which forms of palliative care and how does this differ between regions?

**Method:**

Retrospective cohort study with claims data from insured persons who died in 2016: Based on services billed at least once in the last six months of life, we determined the use of primary palliative care (PPC), specialized palliative homecare (SPHC), as well as inpatient palliative and hospice care, using regional billing codes for PPC and SPHC services for the first time.

**Results:**

Of the 95,962 deceased in the study population, 32.7% received palliative care nationwide, with variations from 26.4% in Bremen to 40.8% in Bavaria. PPC services were billed at 24.4% (16.9% in Brandenburg to 34.1% in Bavaria). SPHC services received 13.1% (6.3% in Rhineland-Palatinate to 18.9% in Brandenburg and 22.9% in Westphalia-Lippe with different SPHC practices). Inpatient palliative care was received by 8.1% (6.7% in Schleswig-Holstein/Hesse to 13.0% in Thuringia); 3.3% (1.6% in Bremen to 5.6% in Berlin) with hospice services.

**Conclusion:**

SPHC is used more frequently than previously reported, while PPC is declining. Utilization seems to be based less on objective needs than on region-specific framework conditions. Besides needs criteria, further development of palliative care should be oriented more towards outcomes and relevant framework conditions.

## Einführung

Das Angebot an Palliativversorgung (PV) in Deutschland wurde in den letzten 20 Jahren stark ausgebaut. Besondere Aufmerksamkeit kam der spezialisierten ambulanten PV (SAPV) zu, die mit dem GKV(gesetzliche Krankenversicherung)-Wettbewerbsstärkungsgesetz 2007 Erstattungsfähigkeit durch die gesetzliche Krankenversicherung erlangte (§ 37b SGB V). Dabei ergänzt sie „… das bestehende Versorgungsangebot, insbesondere das der Vertragsärzte, Krankenhäuser und Pflegekräfte“ (§ 1 Abs. 6 der SAPV-Richtlinie [1]). Gleichzeitig stieg die Anzahl an Hospizen und weitere Palliativstationen an Krankenhäusern wurden eingerichtet. Spezifische Qualifizierungsmaßnahmen für ärztliche und pflegerische Leistungserbringer wurden ausgeweitet und über alle Formen der PV hinweg wurden Leistungen besser vergütet. Dies gilt auch für die allgemeine ambulante PV (AAPV), die v. a. durch Hausärzte^1^, ergänzend auch durch niedergelassene Fachspezialisten, insbesondere Onkologen, getragen wird.

Die Berichterstattung zur Inanspruchnahme von PV am Lebensende wird derzeit im Wesentlichen durch öffentliche Statistiken getragen (SAPV-Frequenzstatistik, Kassenärztliche Bundesvereinigung; KG3-Statistik, DRG[Diagnosis Related Groups]-Statistik, gbe-bund.de). Hierbei fehlt jedoch der Personenbezug: Die SAPV-Frequenzstatistik zählt ausgestellte SAPV-Erst- und Folgeverordnungen personenunabhängig; SAPV-Verordnungen aus dem Krankenhaus, die für bis zu 7 Tage ausgestellt werden können, werden nicht erfasst. Die KG3-Statistik der SAPV-Abrechnungsfälle erfolgt quartalsweise; Fälle, die über Quartalsgrenzen verlaufen, werden in beiden Quartalen gezählt. Auch die DRG-Statistik ist fallbezogen. Die AAPV wurde bisher in keiner öffentlichen Statistik erfasst [2]. Neben der Berichterstattung zur PV für Deutschland insgesamt [3] wurden in einzelnen Bundesländern Gutachten zur regionalen Hospiz- und Palliativversorgung erstellt [4–8].

Auf Versichertenebene wurde die Inanspruchnahme von PV bisher nur aus der Kassenärztlichen Vereinigung (KV) Nordrhein berichtet [9]. In anderen KV-Regionen und bundesweit wurde sie bislang nur von Radbruch et al. analysiert [10]. Die Grundlage bildeten dabei EBM-Ziffern (Einheitlicher Bewertungsmaßstab): Die SAPV-Inanspruchnahme wurde auf Basis der SAPV-Erst- und Folgeverordnungen, die AAPV-Inanspruchnahme auf Basis der Ziffern des EBM-Kapitels „Palliativmedizinische Versorgung“ ermittelt.

Für AAPV-Leistungen und SAPV-Leistungen gibt es neben EBM-Ziffern auch (selektivvertragliche oder KV-spezifische) Sonderziffern, die für die Ermittlung der Inanspruchnahme von PV auf Versichertenebene ebenfalls zu berücksichtigen sind. Zudem können SAPV-Leistungen auch ohne eine in den Abrechnungsdaten dokumentierte Verordnung erbracht werden, z. B. wenn die Verordnung durch einen Krankenhausarzt ausgelöst wurde. Deshalb müssen auch SAPV-Leistungsabrechnungen herangezogen werden, um die Inanspruchnahme palliativer Leistungen möglichst vollständig darzustellen. Hospizleistungsabrechnungen können das Bild weiter vervollständigen.

Die Kenntnis über die tatsächliche Inanspruchnahme palliativer Versorgungsformen ist eine wichtige empirische Grundlage für die Weiterentwicklung der Rahmenbedingungen für eine bedarfsgerechte und wirtschaftliche PV in allen Regionen Deutschlands. Ziel der vorliegenden Arbeit ist es, ein umfassenderes Bild der PV am Lebensende in Deutschland zu geben: Welche Art(en) der PV erhalten GKV-Versicherte am Lebensende und wie variiert dies regional?

## Methoden

Als Teilprojekt des G‑BA-innovationsfondsgeförderten Projekts SAVOIR [11], FKZ 01VSF16005, führten wir eine retrospektive Kohortenstudie mit GKV-Abrechnungsdaten durch, die im wissenschaftlichen Datawarehouse der BARMER bereitgestellt wurden. Primäres Einschlusskriterium für die Studienpopulation war ein Todesdatum im Jahr 2016.

Die Zuordnung der Versicherten zu den Kohorten erfolgte anhand einer mindestens einmaligen Abrechnung einer spezifischen Ziffer innerhalb der letzten 6 Lebensmonate: *AAPV* wurde über die EBM-Ziffern für palliativmedizinische Versorgung (s. EBM, Kapitel 3.2.5.) und zusätzlich über KV-spezifische und selektivvertragliche Sonderziffern identifiziert. *SAPV* identifizierten wir einerseits über EBM-Ziffern der SAPV-Erst- und Folgeverordnung und andererseits über KV-spezifische und selektivvertragliche Sonderziffern sowie SAPV-Leistungsabrechnungen. *Stationäre Palliativversorgung* wurde über die entsprechenden Codes für palliativmedizinische Prozeduren des Operationen- und Prozedurenschlüssels (OPS) identifiziert. *Stationäre Hospizleistungen* (§ 39a Abs. 1 SGB V) wurden anhand von Hospizleistungsabrechnungen erfasst.

Eine detaillierte Beschreibung der Aufgriffskriterien findet sich im Online-Zusatzmaterial zu diesem Beitrag.

Die Daten wurden auf Bundesebene sowie auf Ebene der KV-Region der Versicherten deskriptiv mittels SAS Enterprise Guide Version 7.13 (SAS Institute Inc./Cary/NC/USA) ausgewertet. Alle Ergebnisse wurden standardisiert auf die Alters- und Geschlechtsstruktur aller im Jahr 2016 in Deutschland Verstorbenen (destatis.de).

## Ergebnisse

### Studienpopulation

Insgesamt wurden 95.962 Versicherte (=Verstorbene) eingeschlossen. Das entspricht einem Anteil von 10,7 % aller 2016 in Deutschland Verstorbenen ab 20 Jahre (destatis.de). Die Versicherten waren zum Todeszeitpunkt im Mittel 78,3 ± 13,6 Jahre alt; der Frauenanteil betrug 50,8 %; 29,5 % waren Pflegeheimbewohner. (Zur Verteilung der Studienpopulation über die einzelnen KV-Regionen im Vergleich zur Verteilung aller Verstorbenen auf die Bundesländer siehe Online-Zusatzmaterial, eTab. 1.).

### Inanspruchnahme von Palliativversorgung

32,7 % der Versicherten erhielten in den letzten 6 Lebensmonaten mindestens eine palliative Leistung; ohne Sonderziffern und SAPV- sowie Hospizleistungsabrechnungen, d. h. unter ausschließlicher Berücksichtigung von EBM-Ziffern für AAPV-Leistungen und SAPV-Verordnungen, betrug dieser Anteil 27,1 %. *AAPV* wurde bei 24,4 % der Versicherten abgerechnet, wobei 5,1 % über Sonderziffern identifiziert wurden. 16,5 % der Versicherten erhielten als ambulante PV ausschließlich AAPV (ohne SAPV). *SAPV*-Leistungen nahmen 13,1 % der Versicherten in Anspruch, gut die Hälfte davon identifiziert anhand von SAPV-Erst- und Folgeverordnungen (7,1 %). 5,2 % der Versicherten erhielten nur SAPV (ohne AAPV). Unsere Analysen ergaben, dass bei 20,6 % der Versicherten, die SAPV-Leistungen erhielten, die entsprechende Verordnung im Krankenhaus erfolgte. Bei 8,1 % der Versicherten wurden *stationäre palliativmedizinische Leistungen* abgerechnet; 3,0 % erhielten ausschließlich stationäre palliativmedizinische Leistungen. *Stationäre Hospizleistungen* wurden von 3,3 % der Versicherten in Anspruch genommen (Tab. 1).

**Table 1:** 

Bezugsgrundlage	Anteil Versicherte in den Versorgungsformen [%]			
	AAPV	SAPV	Stationäre PV	Hospiz
*EBM-Ziffern für SAPV-VO sowie AAPV-Leistungen & Krankenhaus-OPS-Codes*	19,3	7,1	8,1	–
*ZUSÄTZLICH regionale Sonderziffern sowie SAPV- und Hospizleistungsabrechnungen*	24,4	13,1	8,1	3,3

Quelle: eigene Berechnungen auf der Grundlage von BARMER-Daten

*AAPV* allgemeine ambulante Palliativversorgung, *SAPV* spezialisierte ambulante Palliativversorgung, *PV* Palliativversorgung, *EBM* Einheitlicher Bewertungsmaßstab, *OPS* Operationen- und Prozedurenschlüssel

^a^Studienpopulation: im Jahr 2016 verstorbene Versicherte der BARMER, *N* = 95.962

### Inanspruchnahme nach KV-Regionen

**Figure 1:**
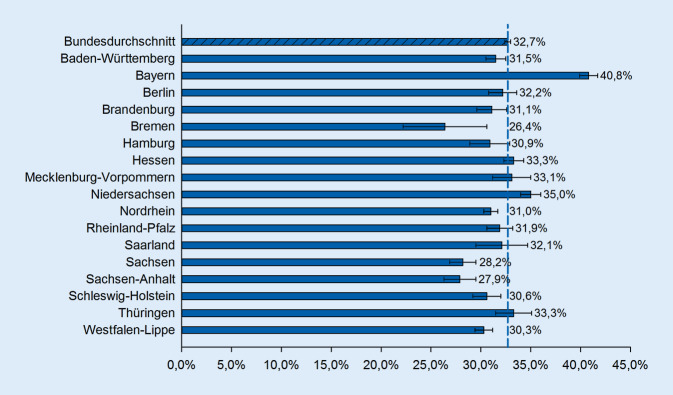

Die Auswertung der Inanspruchnahme von PV auf der Ebene der KV-Region der Versicherten zeigte deutliche regionale Unterschiede (Abb. 1 und 2a–d). In Bayern und in Niedersachsen erhielt im Vergleich zum Bundesdurchschnitt ein hoher Anteil an Versicherten am Lebensende PV, während in Bremen, Sachsen-Anhalt und Sachsen anteilig deutlich weniger Versicherte palliativ versorgt wurden (Abb. 1).

**Figure 2:**
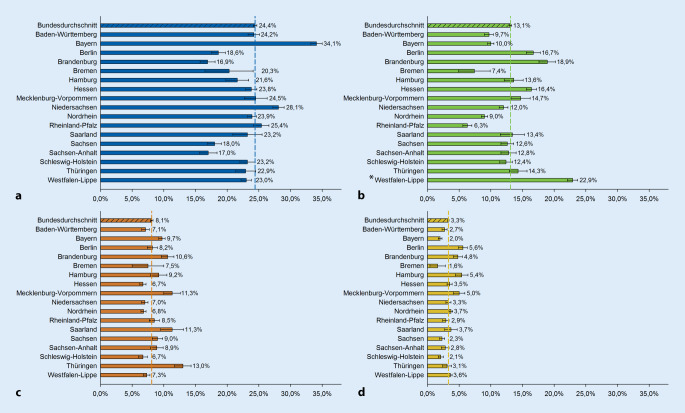

Der Anteil an Versicherten mit *AAPV* war in Bayern mit 34,1 % um fast 10 Prozentpunkte höher als im Bundesdurchschnitt. Auch in Niedersachsen erhielt mit 28,1 % ein vergleichsweise hoher Anteil an Versicherten vor dem Tod AAPV. Im Gegensatz dazu lag der Anteil an Versicherten mit AAPV-Leistungen in Brandenburg (16,9 %), Sachsen-Anhalt (17,0 %), Sachsen (18,0 %) und Berlin (18,6 %) deutlich unter dem Bundesdurchschnitt (Abb. 2a).

Für *SAPV*-Leistungen zeigten sich noch deutlich stärkere Unterschiede zwischen den KV-Regionen. Mit nur 6,3 % war der Anteil an Versicherten mit SAPV in Rheinland-Pfalz weniger als halb so hoch wie im Bundesdurchschnitt. Auch in Bremen (7,4 %), Nordrhein (9,0 %), Baden-Württemberg (9,7 %) und Bayern (10,0 %) erhielten deutlich weniger Versicherte SAPV-Leistungen als im Bundesdurchschnitt. Einen hohen Anteil an Versicherten mit SAPV-Leistungen wiesen hingegen Brandenburg (18,9 %), Berlin (16,7 %) und Hessen (16,4 %) auf. Die Leistungen des Palliativmedizinischen Konsiliardienstes (PKD) in Westfalen-Lippe wurden von 22,9 % der Versicherten in Anspruch genommen (Abb. 2b).

*Stationäre PV* im Krankenhaus wurde von Versicherten der KV-Regionen Thüringen (13,0 %), Mecklenburg-Vorpommern und Saarland (je 11,3 %) sowie Brandenburg (10,6 %) anteilig häufiger und seltener in Schleswig-Holstein, Hessen (je 6,7 %) und Nordrhein (6,8 %) in Anspruch genommen (Abb. 2c).

*Stationäre Hospizleistungen* wurden in Bremen (1,6 %), Bayern (2,0 %) und Schleswig-Holstein (2,1 %) anteilig weniger in Anspruch genommen, während der Anteil in Berlin (5,6 %), Hamburg (5,4 %) und Mecklenburg-Vorpommern (5,0 %) über dem Bundesdurchschnitt lag (Abb. 2d).

## Diskussion

Unsere Analyse liefert ein nach KV-Regionen differenziertes Bild der Palliativversorgung (PV) in Deutschland. Die Inanspruchnahme verschiedener Formen der PV am Lebensende auf Versichertenebene wurde anhand von Krankenkassenabrechnungsdaten quantifiziert. Dabei wurden erstmals auch Sonderziffern und SAPV- sowie Hospizleistungsabrechnungen herangezogen. Die Ergebnisse zeigen, dass deren Berücksichtigung essenziell ist: Der Anteil an Versicherten mit SAPV-Leistungen betrug im Jahr 2016 nicht 7,1 % wie auf Basis der Verordnungen ermittelt, sondern war mit 13,1 % beinahe doppelt so hoch. Und auch dieser Anteil dürfte noch unterschätzt sein, da SAPV-Abrechnungen für die KV Berlin nicht vollständig zur Verfügung standen. Bei etwa einem Fünftel der Versicherten mit SAPV wurde die SAPV im Krankenhaus verordnet – ein großer Anteil, der bislang nicht quantifiziert wurde.

Der Anteil der Versicherten, der in den letzten 6 Lebensmonaten AAPV erhielt, wurde erstmals umfassend geschätzt und beträgt 24,4 %. Er ist aber wegen vermuteter Lücken bei der Identifikation von Vergütungsziffern integrierter Versorgungsverträge sowie einer möglicherweise lückenhaften Dokumentation palliativmedizinischer Leistungen durch Hausärzte ebenfalls eher unterschätzt. Bei der Versorgung älterer, gebrechlicher Patienten sieht das Vergütungssystem nämlich eine Trennung zwischen geriatrischen und palliativmedizinischen Leistungen vor, die in der hausärztlichen Praxis verschmelzen [12], was mit einer Unterdokumentation palliativmedizinischer Leistungen einhergehen kann [13].

### Vergleich mit Bedarfsschätzungen und im zeitlichen Verlauf

Der Anteil von 32,7 % der Versicherten mit mindestens einer Palliativleistung in den letzten 6 Lebensmonaten liegt noch immer deutlich unter dem geschätzten Bedarf für Deutschland: Unter Verwendung der Schätzmethode von Murtagh et al. [14] wiesen 78 % der Todesfälle im Jahr 2013 einen palliativen Versorgungsbedarf auf [15]. Auf internationaler Ebene wird der Bedarf auf 56–83 % geschätzt [16].

Laut Schätzungen der Deutschen Gesellschaft für Palliativmedizin (DGP) haben sogar 90 % der Versterbenden palliativen Versorgungsbedarf, davon wiederum etwa 10 % Bedarf an spezialisierter PV [10, 17]. Diesem geschätzten Bedarf von 90 % bzw. 10 % steht in unseren Analysen eine Inanspruchnahme von 32,7 % bzw. 13,1 % gegenüber. Demnach bestünde insgesamt eine deutliche palliative Unterversorgung bei einer gleichzeitig über der genannten Bedarfsschätzung liegenden Inanspruchnahme von SAPV.

Mit 13,1 % erhielten im Jahr 2016 deutlich mehr Menschen vor ihrem Tod SAPV-Leistungen als bisher berichtet (im Mittel 3,5 % für die Jahre 2010–2014 [10]) bzw. als es auf Basis der SAPV-Verordnungen ermittelbar war (7,1 %). Dies deckt sich mit dem Anstieg der Anzahl an SAPV-Verträgen bundesweit von 261 auf 293 (+12,3 %) zwischen 2014 und 2016 [18]. Die starke Verbreitung der SAPV zeigt sich schließlich auch in der Gegenüberstellung der SAPV-Inanspruchnahme von 13,1 % mit der alleinigen Inanspruchnahme von AAPV von nur 16,5 %.

Der Anteil von Versicherten mit AAPV-Leistungen lag mit 24,4 % im Jahr 2016 auf demselben Niveau wie es von Radbruch et al. [10] für 2014 berichtet wurde. Zieht man aber – wie ebendort – ausschließlich die EBM-Ziffern der AAPV heran, so lag die von uns ermittelte Inanspruchnahme im Jahr 2016 bei 19,3 %. Während also die Inanspruchnahme von SAPV stark zugenommen hat, ist bei AAPV ein Rückgang zu verzeichnen, und dies obwohl der geschätzte Bedarf an PV insgesamt bei Weitem nicht gedeckt ist.

### Regionaler Vergleich

Erkennbar ist eine starke regionale Heterogenität in der Inanspruchnahme unterschiedlicher Formen der PV, hier insbesondere im Verhältnis allgemeiner zu spezialisierter Versorgung.

Potenzielle Einflussfaktoren stellen neben Bedarfsunterschieden und nachfrageseitigen Unterschieden z. B. in der wahrgenommenen Qualität oder Kenntnis der Angebote (bei Patienten/Angehörigen wie auch betreuenden Versorgern) insbesondere regionsspezifische Rahmenbedingungen wie Versorgungskulturen und Leistungs- und Vergütungsbedingungen palliativer Versorgungsformen dar, aus denen regional unterschiedliche Angebotsstrukturen hervorgehen. Für die SAPV gilt dabei eine nur eingeschränkte interregionale Vergleichbarkeit in Leistungsart und -umfang [17] aufgrund unterschiedlicher Vertragsbedingungen [19]. In geringerem Maße gelten solche Unterschiede auch für die AAPV, wenn KV- oder selektivvertragliche Sonderziffern existieren. Der grundsätzlich höhere Spezialisierungs- und Anforderungsgrad ebenso wie eine höhere Vergütung von SAPV-Leistungen gegenüber AAPV-Leistungen ist jedoch durchgängig in allen Regionen vorhanden. Während für jedes Hospiz ein eigener Vergütungssatz (tagesbezogener Bedarfssatz) mit den Kassenverbänden auf Landesebene vereinbart wird [20], werden Palliativleistungen im Krankenhaus überwiegend bundeseinheitlich vergütet (bis auf unterschiedliche Landesbasisfallwerte und bundeslandspezifische Zusatzentgelte für besondere Einrichtungen).

Der Zusammenhang zwischen Inanspruchnahme und Angebotskapazitäten zeigt sich eindrucksvoll in der nahezu exakten Deckung regionaler Inanspruchnahme stationärer PV im Krankenhaus (s. Ergebnisse) mit dem jeweiligen Angebot an Palliativbetten [21]. Beinahe ebenso deutlich deckt sich das Angebot an Hospizbetten [21] mit der Inanspruchnahme stationärer Hospizleistungen (s. Ergebnisse).

Für die jeweilige Entscheidung, ob AAPV oder SAPV zum Einsatz kommt, liegen Hinweise vor, dass diese in hohem Maße von den verfügbaren hausärztlichen AAPV-Kapazitäten abhängt [22]. Weitere Faktoren zur Erklärung regionalspezifischer Inanspruchnahmemuster sollen im Folgenden für ausgewählte KV-Regionen mit niedriger bzw. hoher Inanspruchnahme von AAPV bzw. SAPV erörtert werden:

*Bayern* fällt durch die mit 34,1 % im Bundesvergleich sehr hohe Inanspruchnahme von AAPV auf. Hier gab es bereits sehr früh eine starke ambulante Hospizkultur. Die damit einhergehenden Netzwerke (der Hospiz- und Palliativversorgung) haben die regionale palliative Versorgungslandschaft geprägt [17]. 2011 wurde das „Rahmenkonzept zur Hospiz- und Palliativversorgung“ veröffentlicht [23]. Der Grundsatz „ambulant vor stationär“ sowie die multiprofessionelle Vernetzung auch über Sektorengrenzen hinweg spielen eine wesentliche Rolle. Hinzu kommt, dass die hausarztzentrierte Versorgung (§ 73b SGB V) in Bayern gut etabliert ist und eigene Vergütungsziffern für AAPV-Leistungen vorsieht. Im Vergleich dazu lag die Inanspruchnahme von SAPV unter dem Bundesdurchschnitt. Die SAPV-Teams sind zum Großteil auf Initiative der o. g. Netzwerke bzw. der Hospiz- und Palliativvereine entstanden und fügen sich damit in die vorhandene Netzwerkstruktur ein. Im Vergleich zum Bundesdurchschnitt verfügt Bayern über deutlich weniger Ärzte mit Zusatzweiterbildung „Palliativmedizin“ (Online-Zusatzmaterial, eTab. 2). Dies ist womöglich auf spezifische Anforderungen in der Weiterbildungsordnung der Bayerischen Landesärztekammer für diese Zusatzweiterbildung zurückzuführen. Die Mehrheit der Palliativmediziner ist stationär tätig (eTab. 2).

*Berlin* fällt durch die mit 16,7 % hohe Inanspruchnahme von SAPV auf. Bereits seit 1992 wurden hier Vereinbarungen nach § 73a SGB V für die Präfinalversorgung von Krebs- oder Aidspatienten geschlossen – die sog. Home-Care-Versorgung [24]. Die SAPV baute auf diesen Strukturen auf [24]: Home Care Berlin e. V. (=Berliner Landesverband der Bundesarbeitsgemeinschaft SAPV) koordiniert die in der SAPV tätigen Ärzte, Pflegedienste sowie weitere Leistungserbringer, die in offenen Teams zusammenarbeiten. Dabei dürfen die spezialisierten Palliativärzte SAPV-Folgeverordnungen selbst ausstellen und abrechnen (s. Rahmenvertrag für Berlin aus dem Jahr 2013, § 6 Abs. 4), ggf. auch für Aufgaben, die in anderen Bundesländern der AAPV zugerechnet werden [10]. Dies erklärt die hohe Inanspruchnahme von SAPV in Berlin. Demgegenüber war die Inanspruchnahme von AAPV-Leistungen hier mit 18,6 % auffällig gering. Hausärzte sind deutlich weniger als in anderen Bundesländern in die PV involviert [25]. Oft läuft diese regelrecht an ihnen vorbei: Sobald erstmals SAPV verordnet wurde, werden sie nicht einmal mehr als Verordner benötigt. Verstärkt wird dieser Effekt noch dadurch, dass SAPV-Erstverordnungen auch häufig aus dem Krankenhaus heraus erfolgen. Berlin verfügt darüber hinaus über ein im Vergleich zu anderen Regionen höheres Angebot an Hospizbetten [21], das auch in Anspruch genommen wurde.

*Brandenburg* fällt durch die mit 16,9 % im Bundeslandvergleich geringste Inanspruchnahme von AAPV auf, auch wenn diese auf Landkreisebene in Abhängigkeit von lokalen Angeboten an Hospiz- und Palliativversorgung variiert [26]. Brandenburg hat zum einen im Bundeslandvergleich eine geringe Anzahl an Hausärzten (eTab. 2, [27]) und gleichzeitig eine im Vergleich zum Bundesdurchschnitt deutlich geringere Einwohnerdichte (eTab. 2). So entstehen je Arzt große Versorgungsradien. Der Anteil an Ärzten mit Zusatzweiterbildung „Palliativmedizin“ ist in Brandenburg vergleichsweise hoch (eTab. 2). Diese sind wegen der größeren Versorgungsradien und besseren Vergütung wahrscheinlich eher im Rahmen der SAPV tätig. In Brandenburg erhielt mit 18,9 % ein überdurchschnittlich hoher Anteil an Versicherten SAPV-Leistungen. Fehlende AAPV-Strukturen werden daher vermutlich durch SAPV substituiert: Anstelle einer AAPV, die als solche ausreichend sein könnte, kommen SAPV-Teams zum Einsatz, weil ansonsten gar keine ambulante PV möglich wäre.

In *Hessen* lag die Inanspruchnahme von SAPV mit 16,4 % deutlich über dem Bundesdurchschnitt, während die Inanspruchnahme von AAPV mit 23,8 % in etwa dem Bundesdurchschnitt entsprach. Die SAPV hat sich in Hessen aus den seit 2005 vereinzelt abgeschlossenen Verträgen zur integrierten Versorgung Palliativmedizin entwickelt [28, 29]. SAPV-Teams (Palliative Care Teams) sind zusammen mit Hausärzten, Pflegediensten, Hospizdiensten und Kliniken in Netzwerken organisiert. Hier liegt somit traditionell ein besonderes Augenmerk auf der Entwicklung adäquater integrierter PV-Strukturen, um den Versorgungsbedarf von Patienten zu decken, der die Leistungsfähigkeit der bestehenden Regelversorgung übersteigt, ohne bereits die Komplexität einer SAPV-Versorgung zu begründen [28, 29]. Im Bundesvergleich gehört Hessen zu den Bundesländern mit den meisten ambulant tätigen Palliativmedizinern (eTab. 2); die SAPV ist flächendeckend ausgebaut. Stationäre PV im Krankenhaus wurde hier eher weniger in Anspruch genommen.

*Niedersachsen* fällt durch den mit 28,1 % im Bundeslandvergleich hohen Anteil an Versicherten mit AAPV-Leistungen auf. Seit 2006 sind hier flächendeckend Palliativstützpunkte als Netzwerke der örtlichen Hospizarbeit und PV entstanden, innerhalb derer vorhandene Angebotsstrukturen kooperativ zusammenarbeiten [30, 31]. Die Palliativstützpunkte bündelten die Strukturen der Spezialversorgung, koordinierten deren Angebot und berieten die Leistungserbringer der Basisversorgung [30]. Damit gab es hier bereits gut vernetzte lokale Strukturen, bevor die SAPV bundesweit durch die GKV erstattungsfähig wurde. Die so gewachsenen Palliativstrukturen haben eine breite Verankerung der PV im Bereich der ambulanten ärztlichen Versorgung insgesamt mit sich gebracht. Niedersachsen verfügt im Bundeslandvergleich über die meisten ambulant tätigen Ärzte mit Zusatzweiterbildung „Palliativmedizin“. Auch ist deren Anteil an allen Ärzten mit ambulanter Tätigkeit hier am höchsten (eTab. 2). Lokale Ärztenetze (nach § 87b SGB V) fördern die Qualifizierung ihrer Ärzte zu Palliativmedizinern, bauen SAPV-Teams auf und gründen Palliativstützpunkte. Radbruch et al. sehen den vergleichsweise hohen Anteil an SAPV in Niedersachsen auch darin begründet, dass durch weniger strikte Struktur- und Prozessvorgaben in SAPV-Verträgen mehr Einrichtungen Leistungen der SAPV anbieten können [10]. In unseren Analysen lag die Inanspruchnahme von SAPV in Niedersachsen mit 12,0 % etwas unter dem Bundesdurchschnitt. Stationäre PV im Krankenhaus wurde in Niedersachsen vergleichsweise weniger in Anspruch genommen.

In *Nordrhein* entsprach die Inanspruchnahme von AAPV (s. auch Online-Zusatzmaterial) mit 23,9 % in etwa dem Bundesdurchschnitt, während die von SAPV mit 9,0 % deutlich darunter lag. Hier wurde bereits frühzeitig auf eine flächendeckende Umsetzung der ambulanten palliativmedizinischen und -pflegerischen Versorgung im Sinne eines kooperativen integrativen Versorgungskonzepts gesetzt [32]. Dabei bilden Ärzte mit palliativmedizinischer Basisqualifikation (Absolvierung einer 40-stündigen Kursweiterbildung Palliativmedizin) gemeinsam mit einem ambulanten palliativpflegerischen Dienst die Basis der Versorgung; bei Bedarf kann ein qualifizierter Palliativarzt (QPA, Voraussetzung: Zusatzbezeichnung „Palliativmedizin“) oder ein weiterer Kooperationspartner (z. B. Hospizdienst) hinzugezogen werden [32]. Mit Einführung des gesetzlichen Anspruchs auf SAPV entstanden sogenannte Palliative Care Teams als SAPV-Dienstleister, in denen QPÄ mit ausgebildeten Palliativpflegekräften zusammenarbeiten. Damit ist zwischen der im Rahmen der AAPV anforderbaren Einzelleistung eines QPA [33] und der Teamleistung der SAPV zu differenzieren [34]. Dies erklärt auch die geringere Inanspruchnahme von SAPV: Große Versorgungsanteile entfallen auf die vorgelagerte Spezialisierungsebene des QPA und damit der AAPV. An der Vereinbarung teilnehmende Haus- und Fachärzte mit palliativmedizinischer Basisqualifikation können als koordinierender Arzt fungieren und ebenfalls AAPV-Leistungen abrechnen [33]. Seit Aufnahme von Abrechnungsziffern für die hausärztliche PV in den EBM im 4. Quartal 2013 existiert in Nordrhein innerhalb der AAPV somit ein dreistufiges System aus Basisversorgung (EBM-Ziffern der Palliativversorgung), koordinierenden Ärzten mit palliativmedizinischer Basisqualifikation sowie QPÄ. Stationäre PV im Krankenhaus wurde in Nordrhein eher weniger in Anspruch genommen.

In *Westfalen-Lippe* wurden schon sehr früh und regelhaft spezielle ambulante Palliativstrukturen gefördert, die palliativmedizinischen Konsiliardienste (PKD). Diese wurden später an die bundesweite Einführung der SAPV angepasst. Die „Vereinbarung zur Umsetzung der ambulanten palliativmedizinischen Versorgung von unheilbar erkrankten Patienten im häuslichen Umfeld“ [35] ermöglicht explizit Übergänge zwischen allgemeiner und spezialisierter Versorgung. Der Hausarzt bleibt hier auch in der palliativen Versorgungssituation der zentrale Ansprechpartner [36, 37]. Eingeschriebene Haus- und Fachärzte arbeiten direkt mit dem PKD zusammen, der die spezialisierte palliativmedizinische Versorgung bei Bedarf übernimmt. Eingeschriebene Ärzte können die AAPV-Ziffern dieser Vereinbarung abrechnen, nicht eingeschriebene Ärzte können nur die Ziffern der Regelversorgung (EBM-Ziffern) abrechnen. Das Hinzuziehen des PKD bedarf keiner SAPV-Verordnung nach Muster 63 [35]. Die spezialisierten palliativmedizinischen Leistungen des PKD werden in dieser Studie der SAPV zugeordnet (siehe Online-Zusatzmaterial „eMethoden“). In unseren Analysen war Westfalen-Lippe mit 22,9 % diejenige KV-Region mit der höchsten Inanspruchnahme von SAPV (im Sinne einer Inanspruchnahme von PKD-Leistungen, s. o. g. Vereinbarung). Gleichzeitig erhielten aber auch 23,0 % der Versicherten am Lebensende AAPV-Leistungen. Ausschließlich AAPV-Leistungen (ohne SAPV) erhielt lediglich ein Fünftel aller AAPV-Versorgten. Die Inanspruchnahme stationärer PV im Krankenhaus lag hingegen mit 7,3 % deutlich unter dem Bundesdurchschnitt.

Im Ergebnis lässt sich eine Gruppe an KV-Regionen identifizieren, in denen eine geübte, historisch gewachsene Zusammenarbeit der an der ambulanten PV mitwirkenden Akteure (Niedersachsen, Hessen) bzw. eine starke Verzahnung von allgemeiner und spezialisierter ambulanter Versorgung (Westfalen Lippe, Nordrhein) existiert. In diesen KV-Regionen ist die Inanspruchnahme stationärer PV eher gering. Dies könnte so gedeutet werden, dass starke, gewachsene, später um SAPV ergänzte ambulante Strukturen den palliativen Versorgungsbedarf zu decken vermögen, ohne vermehrt auf stationäre Kapazitäten zurückgreifen zu müssen. Anders gelagert ist der Fall Bayern, wo die gewachsenen allgemeinen ambulanten Strukturen in Kombination mit einer eher geringfügig ausgebauten SAPV die Inanspruchnahme stationärer Strukturen nicht verhindern.

In einer weiteren Gruppe an KV-Regionen überwiegt die SAPV gegenüber der AAPV, entweder weil AAPV-Versorgungslücken bestehen, d. h. nicht genügend Erbringer von AAPV vorhanden sind (Brandenburg), oder weil die spezialisierten Strukturen besonders leicht zugänglich sind und einen Einsatz der Hausärzte in AAPV überflüssig machen (Berlin).

### Limitationen

Die Limitationen dieser Studie sind im Online-Zusatzmaterial zusammengefasst.

## Fazit

Indem Leistungsziffern so umfassend wie möglich einbezogen wurden, stellen wir die Berichterstattung zur Inanspruchnahme der PV auf eine neue Grundlage: Mit 13,1 % erhielten deutlich mehr Menschen vor ihrem Tod SAPV-Leistungen als bisher berichtet und als bisherige Bedarfsschätzungen beziffern. Gleichzeitig ist die Abrechnung von AAPV-Leistungen stagnierend bzw. rückläufig. Für die stationäre PV ist festzustellen, dass vorhandene Palliativ- (und Hospiz‑)Betten auch genutzt werden. Funktionierende ambulante Palliativstrukturen scheinen weniger stationäre Inanspruchnahme zu erfordern. Der insgesamt möglicherweise eher gering zu bewertende Anteil von Versicherten, die irgendeine Form von PV erhalten (32,7 %), erscheint am ehesten erweiterbar durch eine Stärkung der Strukturen allgemeiner PV.

Des Weiteren wird die starke regionale Heterogenität in der Inanspruchnahme der unterschiedlichen PV-Formen deutlich. Diese ist durch Bedarfsunterschiede allein kaum erklärbar. Vielmehr scheinen regionalspezifische Rahmenbedingungen wie historisch herausgebildete Versorgungskulturen sowie Leistungs- und Vergütungsbedingungen jeweils regionale Angebotsstrukturen hervorgebracht zu haben, die dann auch in Anspruch genommen werden. Regional unterschiedliche Strukturen sind nicht per se negativ. Bewertet werden sollten diese anhand ihrer Outcomes. Diese zu definieren, zu erfassen und zu vergleichen, ist Aufgabe zukünftiger Versorgungsforschung. Gegebenenfalls lassen sich dabei Best-Practice-Regionen identifizieren, deren Wirkelemente sich auch in anderen Regionen implementieren lassen.

Unsere anhand von verfügbaren Regionaldaten angestellte Untersuchung zur Erklärung der regionalen Inanspruchnahme palliativer Versorgungsformen sollte zukünftig anhand von schließenden statistischen Analysen überprüft werden, in denen (geschätzte) Bedarfe sowie weitere nachfrage- und angebotsseitige Einflussfaktoren als unabhängige Größen Berücksichtigung finden. Dennoch lässt sich bereits jetzt schlussfolgern: Die Inanspruchnahme palliativer Versorgungsformen, vor allem aber eine Aufnahme in die allgemeine bzw. spezialisierte Versorgung, ist nicht allein durch medizinischen Bedarf erklärt. Deshalb sollte die Weiterentwicklung der PV zukünftig nicht ausschließlich an Bedarfskriterien, sondern mehr an patientenrelevanten Outcomes, auch in Relation zu den aufgewendeten Kosten, sowie dafür relevanten Rahmenbedingungen auf regionaler wie auf Bundesebene orientiert werden.

## Caption Electronic Supplementary Material



## Footnotes

1 Für eine bessere Lesbarkeit wird nur eine sprachliche Form genutzt; hierbei sind Angehörige aller Geschlechter eingeschlossen.
