# Supplementary material for: Palliative care at the end of life in Germany: Utilization and regional distribution
Source: Bundesgesundheitsblatt Gesundheitsforschung Gesundheitsschutz. 2020 Nov 13;63(12):1502–10. [Article in German] doi: 10.1007/s00103-020-03240-6 (PMC7686196; doi:10.1007/s00103-020-03240-6)
Supplement: Supplementary file 1 [file 103_2020_3240_MOESM1_ESM.pdf]

Bundesgesundheitsblatt 12/2020

Elektronisches Zusatzmaterial zum Beitrag:

Palliativversorgung am Lebensende in Deutschland. Inanspruchnahme und regionale Verteilung

Bianka Ditscheid[1], Markus Krause[1], Thomas Lehmann[2], Kathleen Stichling[1], Maximiliane Jansky[3], Friedemann Nauck[3], Ulrich Wedding[4], Werner Schneider[5], Ursula Marschall[6], Winfried Meißner[4], Antje Freytag[1] für die SAVOIR-Studiengruppe

[1] Institut für Allgemeinmedizin, Universitätsklinikum Jena, Deutschland
[2] Zentrum für Klinische Studien, Universitätsklinikum Jena, Deutschland
[3] Klinik für Palliativmedizin, Universitätsmedizin Göttingen, Deutschland
[4] Abteilung Palliativmedizin der Klinik für Innere Medizin II, Universitätsklinikum Jena, Deutschland
[5] Zentrum für Interdisziplinäre Gesundheitsforschung, Universität Augsburg, Deutschland
[6] BARMER, Wuppertal, Deutschland

Korrespondenzadresse:
Dr. Antje Freytag
Institut für Allgemeinmedizin,
Universitätsklinikum Jena,
Bachstr. 18, 07743 Jena, Deutschland
antje.freytag@med.uni-jena.de

Inhalt:

eMETHODEN: Ergänzungen zur Methode
- Datenquelle
- Ein- und Ausschlusskriterien
- Kohortenzuordnung

eERGEBNISSE: Ergänzungen zu den Ergebnissen
eTab. 1: Verteilung der Studienpopulation auf die Bundesländer und Vergleich mit Daten des Statistischen Bundesamts aus dem Jahr 2016

eDISKUSSION: Ergänzungen zur Diskussion
eTab. 2: Daten verschiedener öffentlicher Statistiken aus dem Jahr 2016
- **Limitationen**

LITERATUR

eMETHODEN: **Ergänzungen zur Methode**

Datenquelle

Für die Analysen wurden pseudonymisierte Versicherten-Stammdaten (Geburtsjahr, Geschlecht, KV (Kassenärztliche Vereinigung)-Region, Versicherungsbeginn, Versicherungsende, Austrittsgrund) und Leistungsdaten aus den Bereichen ambulante vertragsärztliche Versorgung (§§ 294, 295 SGB V) sowie Krankenhaus (§ 301 SGB V) herangezogen. Zusätzlich standen SAPV-Leistungsabrechnungen sowie Abrechnungen stationärer Hospize zur Verfügung. Das positive Votum der Ethikkommission des Universitätsklinikums Jena wurde eingeholt (Bearbeitungsnummer 5317-10/17).

Ein- und Ausschlusskriterien

Das Todesdatum wurde aus den beiden Datenfeldern ‚Versicherungsende' und ‚Austrittsgrund' (Ausprägung: Tod) ermittelt. Weitere Einschlusskriterien waren eine mindestens zweijährige, durchgängige Versicherung bei der datenbereitstellenden Krankenkasse sowie ein Alter von mind. 19 Jahren zum Zeitpunkt des Todes. Ausgeschlossen wurden Versicherte der Deutschen BKK, deren Daten nach der Fusion der BARMER mit der Deutschen BKK zunehmend ins wissenschaftliche Datawarehouse einfließen, aber noch unvollständig sind sowie Versicherte mit fehlender Wohnortinformation oder fehlender KV-Angabe.

Kohortenzuordnung

Allgemeine ambulante Palliativversorgung (***AAPV)*** wurde über die Gebührenordnungspositionen (GOP) des Einheitlichen Bewertungsmaßstabs (EBM) für Ärzte[1] 03370/04370 (Palliativmedizinische Ersterhebung des Patientenstatus inkl. Behandlungsplan), 03371/04371 (Zuschlag zu der Versichertenpauschale 03000/04000 für die palliativmedizinische Betreuung des Patienten in der Arztpraxis), 03372/04372 (Zuschlag zu den Gebührenordnungspositionen 01410 oder 01413 für die palliativmedizinische Betreuung in der Häuslichkeit) und 03373/04373 (Zuschlag zu den Gebührenordnungspositionen 01411, 01412 oder 01415 für die palliativmedizinische Betreuung in der Häuslichkeit) erfasst. Diese GOP sind nur für Haus- und Kinderärzte abrechnungsfähig. Sonderziffern, wie z.B. die von Onkologen (laut Onkologie-Vereinbarung) abrechenbare Palliativziffer oder KV-spezifische bzw. selektivvertragliche Abrechnungsziffern (SGB V §§ 73b, 73c, 140a) wurden recherchiert[2] und ebenfalls für die Identifizierung von AAPV-Leistungen herangezogen.

Aufgrund der besonderen Strukturen für die ambulante Palliativversorgung in Nordrhein und Westfalen-Lippe müssen die von uns verwendeten Zuordnungsregeln für AAPV (und SAPV (Spezialisierte ambulante Palliativversorgung)) explizit erwähnt werden: In der KV Nordrhein gelten sowohl die Leistungen betreuender/koordinierender (Haus)Ärzte als auch die der qualifizierten Palliativärzte (QPÄ) als AAPV-Leistungen [1]. Im Gegensatz dazu werden in der KV-Region Westfalen-Lippe lediglich die Leistungen der teilnehmenden Haus- und Fachärzte als AAPV-Leistungen angesehen [2]. (Palliativmedizinisch qualifizierte Ärzte werden im Rahmen von Palliativmedizinischen Konsiliardiensten (PKD) tätig und werden hier der spezialisierten palliativmedizinischen Versorgung zugeordnet, siehe Abschnitt zur SAPV [2].)

[1] Für eine bessere Lesbarkeit wird nur eine sprachliche Form genutzt; hierbei sind Angehörige aller Geschlechter eingeschlossen.
[2] Die Recherche dieser Ziffern erfolgte anhand der Internet-Auftritte der Kassenärztlichen Vereinigungen, die z.T. durch Anschreiben und Telefonate ergänzt wurden. Auch durch die BARMER wurden Ziffern bereitgestellt.

Die sog. BQKPmV (Besonders Qualifizierte und Koordinierte Palliativmedizinische Versorgung), bei der Ärzte mit entsprechendem Qualifikationsnachweis zur Abrechnung zusätzlicher Vergütungsziffern berechtigt sind [s. Kapitel 37.3 des EBM „BQKPmV“ gemäß Anlage 30 zum BMV-Ä], wurde nicht betrachtet, da diese erst zum 01.10.2017 und somit nach dem in der vorliegenden Studie betrachteten Beobachtungszeitraum eingeführt wurde.
Palliativpflegerische Leistungen wurden nicht erfasst, da eine Abgrenzung von sonstigen Pflegeleistungen in den Abrechnungsdaten (noch) nicht möglich war (Häusliche Krankenpflege HKP Nr. 24a erst 2017 eingeführt).

SAPV (SGB V § 132d i.V.m. § 37b) wurde einerseits über die GOP 01425 für die SAPV-Erstverordnung bzw. 01426 für die SAPV-Folgeverordnung identifiziert. Außerdem wurden auch hier KV-spezifische Sonderziffern recherchiert[3] und erstmals SAPV-Leistungsabrechnungen für die Identifizierung genutzt.
In Westfalen-Lippe ist der PKD der Leistungserbringer der spezialisierten palliativmedizinischen Versorgung [2]. Da die strukturellen Anforderungen an einen PKD von denen an SAPV-Leistungserbringer anderer KV-Regionen abweichen, ist der PKD nicht 1:1 mit der SAPV gleichzusetzen. Hervorzuheben ist auch, dass aufgrund der jeweils lokal gültigen Vereinbarungen [1–3] in den KV-Regionen Nordrhein und Westfalen-Lippe die Leistungen qualifizierter Palliativärzte einmal der AAPV (Nordrhein) und einmal dem PKD (Westfalen-Lippe) zugeordnet werden.
Aufgrund der Einbeziehung von SAPV-Leistungsabrechnungen konnten erstmals auch diejenigen Versicherten identifiziert werden, für die keine SAPV-Verordnung in den Abrechnungsdaten dokumentiert wurde. Das sind im Wesentlichen diejenigen Versicherten, deren SAPV-Verordnung von einem Krankenhaus-Arzt ausgestellt wurde (für max. 7 Tage möglich, siehe SAPV-Richtlinie §7 Abs. 1, Satz 2 u. 3) und die keine Folgeverordnung erhielten. Auch ist es möglich, dass die Ziffer für die SAPV-Verordnung nicht dokumentiert und abgerechnet wird und sie deshalb in den Krankenkassen-Routinedaten nicht vorliegt. So ist in Westfalen-Lippe für das Hinzuziehen des PKD keine SAPV-Verordnung nach Muster 63 erforderlich [2].
Generell konnten nur digital vorliegende SAPV-Leistungsabrechnungen, d.h. keine Papierabrechnungen ausgewertet werden. Der Anteil der SAPV-Verordnungen aus dem Krankenhaus wurde deshalb für Versicherte mit SAPV-Abrechnungen ohne SAPV-Verordnung unter Ausschluss der Versicherten aus den KV-Regionen Westfalen-Lippe (keine SAPV-Verordnung notwendig) und Berlin (SAPV-Leistungsabrechnungen unvollständig) ermittelt. Über Plausibilitätsprüfungen zu Krankenhausaufenthalten vor Beginn der SAPV wurde derjenige Anteil an Versicherten ermittelt, dessen SAPV innerhalb von 7 Tagen nach Entlassung aus dem Krankenhaus begann.
Während (palliativ)pflegerische Leistungen im Rahmen der AAPV nicht ausgewiesen wurden (siehe Abschnitt zur AAPV), können SAPV-Leistungen sowohl ärztliche als auch pflegerische Leistungen enthalten, wobei in einigen SAPV-Verträgen die zusätzliche Verordnung von HKP erlaubt ist und in anderen nicht.

Die ***stationäre Palliativversorgung*** wurde über die Krankenhaus-OPS (Operationen- und Prozedurenschlüssel)-Codes 8-982 (Palliativmedizinische Komplexbehandlung) und 8-98e (Spezialisierte stationäre palliativmedizinische Komplexbehandlung (mit kontinuierlicher, 24-stündiger Behandlung auf einer eigenständigen Palliativeinheit)) zugeordnet. Die

[3] Die Recherche dieser Ziffern erfolgte anhand der Internet-Auftritte der Kassenärztlichen Vereinigungen, die z.T. durch Anschreiben und Telefonate ergänzt wurden. Auch durch die BARMER wurden Ziffern bereitgestellt.

palliativmedizinischen Leistungen „besonderer Einrichtungen“, die anhand von dafür eingeführten Zusatzentgelten abgerechnet werden können, wurden nicht berücksichtigt. Der OPS-Code für den palliativmedizinischen Konsiliardienst OPS 8-98h (Spezialisierte palliativmedizinische Komplexbehandlung durch einen Palliativdienst) wurde erst 2017 eingeführt und wird deshalb hier nicht betrachtet.

eERGEBNISSE: Ergänzungen zu den Ergebnissen

eTab. 1: **Verteilung der Studienpopulation[a] auf die Bundesländer und Vergleich mit Daten des Statistischen Bundesamts aus dem Jahr 2016**

Bundesland[b] (alphabetische Reihenfolge)	Studienpopulation		Verstorbene im Bundesland[c] (Destatis)		Differenz aus Studienpopulation und Destatis	
	Anzahl	*Anteil [%]*	Anzahl	*Anteil [%]*	*absolute Diff. [%]*	*relative Diff. [%]*
**Baden-Württemberg**	7.988	*8,3*	105.972	*11,8*	*-3,5*	*-29,7*
**Bayern**	11.897	*12,4*	128.801	*14,3*	*-1,9*	*-13,3*
**Berlin**	4.403	*4,6*	33.840	*3,8*	*0,8*	*21,1*
**Brandenburg**	3.468	*3,6*	30.661	*3,4*	*0,2*	*5,9*
**Bremen**	422	*0,4*	17.761	*2,0*	*-1,6*	*-80,0*
**Hamburg**	2.033	*2,1*	17.160	*1,9*	*0,2*	*10,5*
**Hessen**	8.272	*8,6*	63.760	*7,1*	*1,5*	*21,1*
**Mecklenburg-Vorpommern**	2.162	*2,3*	20.371	*2,3*	*0*	*0,0*
**Niedersachsen**	8.787	*9,2*	63.760	*7,1*	*2,1*	*29,6*
**Nordrhein-Westfalen[d]**	26.051	*27,1*	201.087	*22,3*	*4,8*	*21,5*
**Rheinland-Pfalz**	5.071	*5,3*	58.871	*6,5*	*-1,2*	*-18,5*
**Saarland**	1.223	*1,3*	12.840	*1,4*	*-0,1*	*-7,1*
**Sachsen**	4.451	*4,6*	53.160	*5,9*	*-1,3*	*-22,0*
**Sachsen-Anhalt**	2.777	*2,9*	31.350	*3,5*	*-0,6*	*-17,1*
**Schleswig-Holstein**	4.505	*4,7*	33.720	*3,7*	*1*	*27,0*
**Thüringen**	2.452	*2,6*	28.214	*3,1*	*-0,5*	*-16,1*
**Gesamt**	**95.962**	***100,0***	**901.328**	***100,0***		

[a] Studienpopulation: im Jahr 2016 verstorbene Versicherte der BARMER, N=95.962
[b] in Studienpopulation KV-Region des Verstorbenen. Die KV-Regionen entsprechen den Bundesländern mit Ausnahme von Nordrhein-Westfalen, das in die KV Nordrhein und die KV Westfalen-Lippe unterteilt ist. Die Verstorbenen unserer Studienpopulation aus diesen KV-Regionen wurden hier zusammengefasst.
[c] ab 20 Jahre, als Anteil an allen Verstorbenen (bundesweit, ab 20 Jahre)
[d] als Summe aus Nordrhein und Westfalen-Lippe

eDISKUSSION: Ergänzungen zur Diskussion

eTab. 2: Daten verschiedener öffentlicher Statistiken aus dem Jahr 2016[a]

Bundesland	Einwohner je km²	Hausärzte je 100.000 Einwohner	Ambulant tätige Palliativmediziner[b]			Palliativmediziner[b] (mit ärztlicher Tätigkeit) je 100.000 Einwohner[c]		
			gesamt[d]	Palliativmediziner[b] (Anzahl)	Palliativmediziner[b] (%)[e]	gesamt	ambulant	stationär
**Baden-Württemberg**	306	66,1	19.599	841	*4,3*	14,0	7,7	5,7
**Bayern**	183	71,6	26.564	313	*1,2*	6,5	2,4	3,6
**Berlin**	4.012	72,6	8.608	142	*1,6*	8,9	4,0	4,1
**Brandenburg**	84	64,4	3.838	179	*4,7*	11,7	7,2	4,3
**Bremen**	1.617	67,2	1.652	28	*1,7*	11,3	4,1	6,3
**Hamburg**	2.397	73,8	4.889	105	*2,1*	11,1	5,8	4,7
**Hessen**	294	65,1	10.784	524	*4,9*	17,3	8,4	7,1
**Meckl.-Vorpommern**	69	72,9	2.705	79	*2,9*	10,8	4,9	5,3
**Niedersachsen**	167	64,7	13.393	817	*6,1*	16,5	10,3	5,5
**Nordrhein-Westfalen**	524	k.A.#	32.000	1.167	*3,6*	14,0	6,5	7,2
**Rheinland-Pfalz**	205	66,5	7.004	255	*3,6*	13,1	6,3	5,9
**Saarland**	388	67,7	1.903	53	*2,8*	10,7	5,3	4,8
**Sachsen**	221	66,3	6.757	203	*3,0*	10,3	5,0	5,1
**Sachsen-Anhalt**	109	64,4	3.432	99	*2,9*	9,3	4,4	4,9
**Schleswig-Holstein**	182	69,6	5.301	272	*5,1*	15,7	9,4	5,6
**Thüringen**	133	68,4	3.560	80	*2,2*	8,9	3,7	5,1
**Deutschland**	**231**	**k.A.**	**151.989**	**5.157**	***3,4***	**12,4**	**6,2**	**5,6**

[a] Einwohner je km²: Statistisches Bundesamt (www.gbe-bund.de); Hausärzte je 100.000 Einwohner: Versorgungsatlas [4]; ambulant tätige Ärzte und Palliativmediziner: Ärztestatistik, Bundesärztekammer
[b] Ärzte mit Zusatz-Weiterbildung „Palliativmedizin"
[c] eigene Berechnung aus absoluten Anzahlen bezogen auf die Anzahl der Einwohner
[d] gesamt = Gesamtanzahl ambulant tätiger Ärzte aller Fachrichtungen
[e] eigene Berechnung (Anteil Palliativmediziner an ‚gesamt' [Gesamtanzahl ambulant tätiger Ärzte aller Fachrichtungen])

Limitationen

Auf die Limitation der Untererfassung palliativmedizinischer Leistungen wurde bereits hingewiesen. Außerdem sind die Studienergebnisse von eingeschränkter Generalisierbarkeit: Wegen des unterschiedlichen Anteils der BARMER-Versicherten in den einzelnen KV-Regionen und der potenziellen Spezifika der durch die BARMER geschlossenen Selektivverträge zur Palliativversorgung, sind die Studienergebnisse trotz der vorgenommenen Alters- und Geschlechtsstandardisierung nur eingeschränkt auf die gesamte GKV (Gesetzliche Krankenversicherung)-Population übertragbar. Die Zuordnung der Versicherten zu den Versorgungsformen erfolgte anhand einmaliger Abrechnung einer entsprechenden Ziffer in den letzten 6 Lebensmonaten; die Kontinuität der Versorgung wurde nicht erfasst. Es konnten nur die SAPV-Abrechnungen herangezogen werden, die in elektronischer Form vorlagen; SAPV-Abrechnungen in Papierform waren nicht auswertbar. Diese Limitation macht sich insbesondere bei der KV-Region Berlin bemerkbar. Insgesamt wurde damit die Inanspruchnahme von SAPV unterschätzt. Auch für die AAPV kann davon ausgegangen werden, dass diese tendenziell

unterdokumentiert ist (z.B. durch die fehlende Abrechnung palliativmedizinischer Ziffern bzw. geriatrischer Pauschalen anstelle von AAPV-Ziffern). Im Bereich der stationären Palliativversorgung wurden palliativmedizinische Leistungen „besonderer Einrichtungen“, die anhand von dafür eingeführten Zusatzentgelten abgerechnet werden können, nicht erfasst. Während stationäre Hospizleistungen erfasst wurden, konnte die Versorgung durch ambulante Hospizdienste nicht berücksichtigt werden, da diese in den Krankenkassen-Routinedaten nicht auf Versichertenebene dokumentiert werden. Die Zuordnung palliativer Leistungen erfolgte anhand der KV-Region der Versicherten unabhängig davon, wo die Leistung erbracht wurde.

Literatur

1. KV Nordrhein (2015) Vereinbarung über die palliativmedizinische Versorgung von unheilbar erkrankten Versicherten im häuslichen Umfeld. Ersatzkassen
2. KV Westfalen-Lippe (2013) Vereinbarung zur Umsetzung der ambulanten palliativmedizinischen Versorgung von unheilbar erkrankten Patienten im häuslichen Umfeld
3. KV Nordrhein (2009) Vertrag über die Erbringung Spezialisierter ambulanter Palliativversorgung (SAPV) in Nordrhein. Ersatzkassen
4. Kassenärztliche Bundesvereinigung — Dezernat Versorgungsmanagement (2019) Vertragsärzte und -psychotherapeuten je 100.000 Einwohner nach Fachgruppen und Regionen für die Jahre 2014 bis 2018 – Statistische Informationen aus dem Bundesarztregister der Kassenärztlichen Bundesvereinigung. Versorgungsatlas-Bericht Nr. 19/09, Berlin
